# Reference values for the left ventricular wall thickness in cardiac MRI in a modified AHA 17-segment model

**DOI:** 10.1186/1532-429X-14-S1-P223

**Published:** 2012-02-01

**Authors:** Florian Andre, Stephanie Lehrke, Hugo A Katus, Henning Steen

**Affiliations:** 1 Department of Cardiology, University of Heidelberg, Heidelberg, Germany

## Background

Left ventricular (LV) myocardial wall thickness is influenced by non-ischemic conditions like cardiomyopathies (CMP) as well as ischemic disorders, i. e. coronary artery disease (CAD). For CMP, regional wall thickness under resting conditions has prognostic value and serves as risk predictor for future cardiovascular events. In CAD, aside from perfusion also inadequate dobutamine wall thickening indicates myocardial ischemia and leads to coronary intervention. Therefore, quantitative resting and stress regional wall thickness provides relevant diagnostic and prognostic information. To date there is only scarce data regarding reference values of regional wall thickness in healthy volunteers.

We sought to investigate differences in age and gender of LV rest and stress regional wall thickness with a state-of-the-art SSFP sequence in a modified AHA 17-segment model and present reference values for myocardial rest and stress wall thickness.

## Methods

We studied 119 healthy volunteers in two gender groups (60 male, 56 female) which both consisted to equal parts of three age groups to minimize its possible influence. 30 of the male and 29 of the female participants were pharmacologically stressed with dobutamine up to their age-depended maximal heart rate according to present guidelines. Long axis were obtained on a 1.5T whole body MRI scanner (Philips Achieva) using the SSFP sequence. Images of the left ventricle were analyzed applying a modified 17-segment model. P<0.05 was considered significant.

## Results

**Figure 1:**
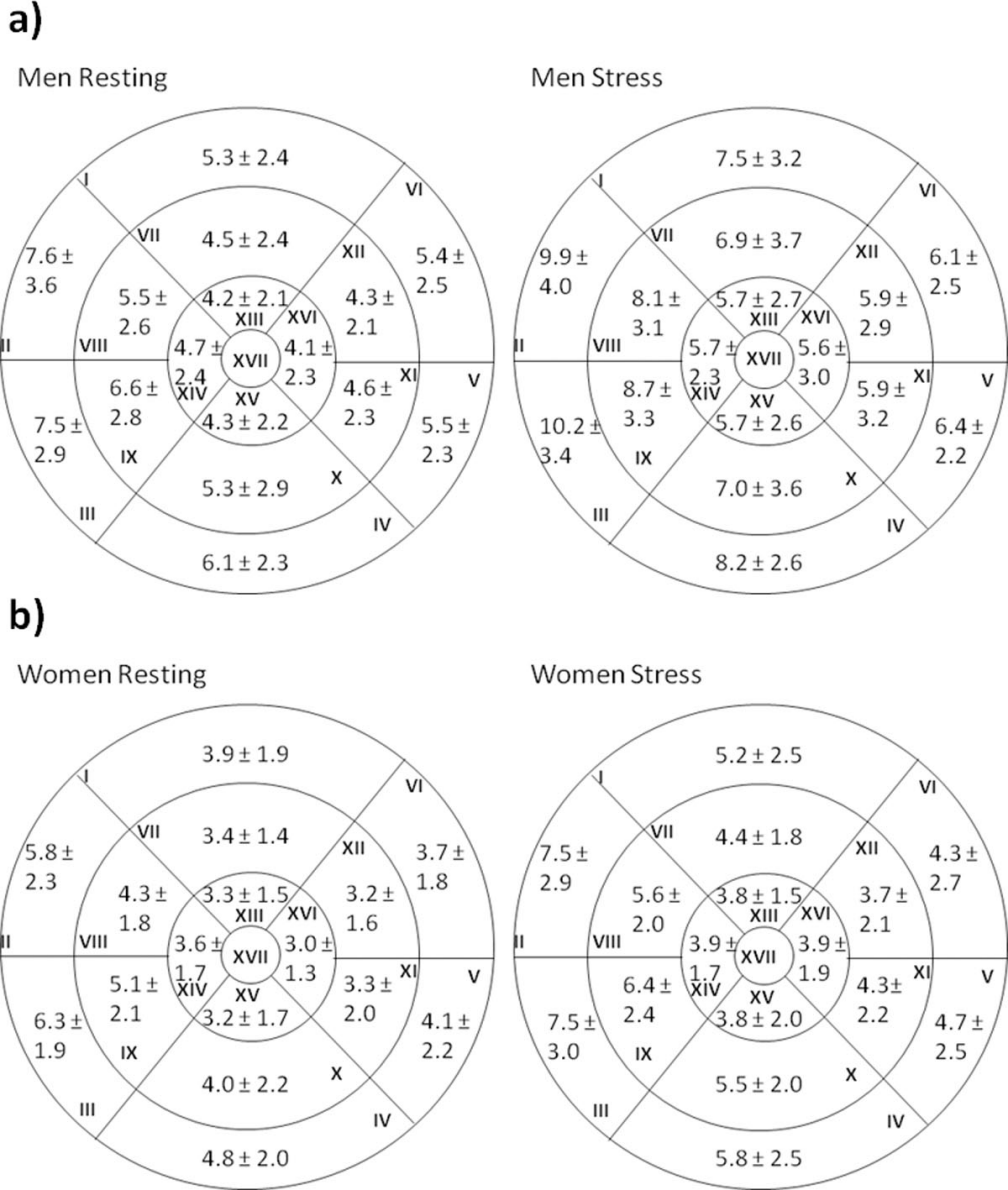
All values are given in mm ± 2 standard deviations.

The acquired normal values are shown in figure 1a and 1b. Both gender groups are similar in age (42.1±12.1 yrs vs. 41.8±13.3, p>0.9).The wall thickness of each segment differs significantly between men and women at rest as well as during stress (both p<0.001). Furthermore the discrepancies of the wall thickness between resting and stress in both gender groups were significant in each segment except of the apic-septal one in women.

## Conclusions

Applying a state-of-the-art SSFP MRI sequence in three different age groups of male and female healthy volunteers, we provide reference values for myocardial LV wall thickness at rest and under dobutamine stress which could lead to a clinical reference frame for the detection of abnormal myocardial thickness in CMP or thickening in CAD. The values for each segment of the left ventricle are significantly different between men and women as well as rest and stress. Further investigations with larger patient cohorts may address the issue of age dependency.

## Funding

None.

